# Synthesis, crystal structure determination of a novel phosphate Ag_1.64_Zn_1.64_Fe_1.36_(PO_4_)_3_ with an alluaudite-like structure

**DOI:** 10.1107/S2056989020011408

**Published:** 2020-08-25

**Authors:** Jamal Khmiyas, Abderrazzak Assani, Mohamed Saadi, Lahcen El Ammari

**Affiliations:** a Laboratoire de Chimie Appliquée des Matériaux, Centre des Sciences des Matériaux, Faculty of Sciences, Mohammed V University in Rabat, Avenue Ibn Batouta, BP 1014, Rabat, Morocco

**Keywords:** orthophosphate, alluaudite-like structure, disorder, X-ray diffraction, crystal structure

## Abstract

Single crystals of Ag_1.64_Zn_1.64_Fe_1.36_(PO_4_)_3_ [silver zinc iron phosphate (1.64/1.64/1.36/3)] have been synthesized by a conventional solid-state reaction and structurally characterized by single-crystal X-ray diffraction. The title compound crystallizes with an alluaudite-like structure. All atoms of the structure are in general positions except for four, which reside on special positions of the space group, *C*2/*c*. The Ag^+^ cations reside at full occupancy on inversion centre sites and at partial occupancy (64%) on a twofold rotation axis. In this structure, the unique Fe^3+^ ion with one of the two Zn^2+^ cations are substitutionally disordered on the same general position (Wyckoff site *8f*), with a respective ratio of 0.68/0.32 (occupancies were fixed so as to ensure electrical neutrality for the whole structure). The remaining O and P atoms are located in general positions. The three-dimensional framework of this structure consists of kinked chains of edge-sharing octa­hedra stacked parallel to [10]. These chains are built up by a succession of [*M*O_6_] (*M* = Zn/Fe or Zn) units. Adjacent chains are connected by the PO_4_ anions, forming sheets oriented perpendicular to [010]. These inter­connected sheets generate two types of channels parallel to the *c* axis, in which the Ag^+^ cations are located. The validity and adequacy of the proposed structural model of Ag_1.64_Zn_1.64_Fe_1.36_(PO_4_)_3_ was established by means of bond-valence-sum (BVS) and charge-distribution (CHARDI) analysis tools.

## Abstract 2

The orthophosphate, Ag_1.64_Zn_1.64_Fe_1.36_(PO_4_)_3_ crystallizes in an alluaudite-type structure. The chains characterizing the alluaudite structure are then built up from edge-sharing [Fe/Zn_6_] and [ZnO_6_] octa­hedra linked together by PO_4_ tetra­hedra. The Ag+ are located in channels parallel to the *c* axis.

## Chemical context   

The first crystal structure of natural alluaudite was determined by Fisher (1955 ▸) using a specimen of pegmatite from Buranga-Rwanda. The metallic monophosphates belonging to this large alluaudite family form an important class of materials whose numerous phases present rich chemistry and great structural originality. Moore (1971 ▸) proposed the following general formulation for alluaudites: *A*(2)*A*(1)*M*(1)*M*(2)_2_(PO_4_)_3_ with *A* and *M* being cationic sites classified in decreasing order of size (*r_M_*_(2)_<*r_M_*_(1)_ <*r_A_*_(1)_<*r_A_*_(2)_). In this structure, the first site *A*(1) can host a mono- or divalent cation and a vacancy (□), while the second site, *A*(2) contains a vacancy (□) as well as a monovalent cation (Moore & Ito, 1979 ▸). The other sites, *M*(1) and *M*(2), display octa­hedral geometries, which may contain a distribution of di- and trivalent cations. The natural alluaudite studied by Moore exhibits the following chemical formula: Na_2.5_Li_0.1_Ca_0.5_Mn_4.5_^2+^Mg_0.2_Fe_7.9_^3+^(PO_4_)_12_ and crystallizes in the monoclinic system, space group *C*2/*c*. In the structure of this compound, the cations are distributed over the four types of site as follows: *A*(1): 2.5Na^+^ + 0.7Mn^2+^ + 0.5Ca^2+^ + 0.3□, *A*(2): 4□, *M*(1): 3.8Mn^2+^ + 0.1Mg^2+^ + 0.1Li^+^, *M*(2): 7.9Fe^3+^ + 0.1Mg^2+^. Later, Hatert *et al.* (2000 ▸) proposed a complex and more accurate general formula for the alluaudite structure in order to take into account the different cationic sites available within the channels in the structure.

The main characteristic of the alluaudite structure is the remarkable flexibility of its anionic framework, which is amenable to various cationic substitutions in the *A* and *M* sites (Chaalia *et al.*, 2012 ▸). As a result, a large number of alluaudite compounds with inter­esting physical properties have been synthesized and systematically characterized. Indeed, the existence of transition metals in the structure is often the origin of inter­esting properties *viz.* magnetic (Hatert *et al.*, 2004 ▸), heterogeneous catalysis [*e.g.*, the role of AgCaCdMg_2_(PO_4_)_3_ and AgCd_2_Mg_2_(PO_4_)_3_ in the conversion of butan-2-ol] (Kacimi *et al.*, 2005 ▸), electronic conductivity and significant ionic mobility (Richardson, 2003 ▸).

Accordingly, our efforts have mainly focused on the development and characterization of new alluaudite-type phosphates in *M*_2_O–*M*′O–P_2_O_5_ systems (*M* = monovalent cation, *M*′ = divalent cation). The hydro­thermal study of the pseudo-ternary system Na_2_O–MgO–P_2_O_5_ allowed the isolation of the alluaudite based on sodium and magnesium: NaMg_3_(PO_4_)(HPO_4_)_2_ (Ould Saleck *et al.*, 2015 ▸). Similarly, the investigation of the two pseudo-quaternary systems Na_2_O–CoO–Fe_2_O_3_–P_2_O_5_ and Na_2_O–ZnO–Fe_2_O_3_–P_2_O_5_, made it possible to obtain two new phases: Na_2_Co_2_Fe(PO_4_)_3_ (Bouraima *et al.*, 2015 ▸) and Na_1.67_Zn_1.67_Fe_1.33_(PO_4_)_3_ (Khmiyas *et al.*, 2015 ▸), by a solid-state route. Herein we report the synthesis of the new phosphate Ag_1.64_Zn_1.64_Fe_1.36_(PO_4_)_3_ and its structural characterization by single crystal X-ray diffraction. The suggested structural model is supported by means of bond-valence-sum (BVS) (Altermatt & Brown, 1985 ▸) and charge-distribution (CHARDI) (Nespolo *et al.*, 2001 ▸) validation methods.

## Structural commentary   

**Figure 1:**
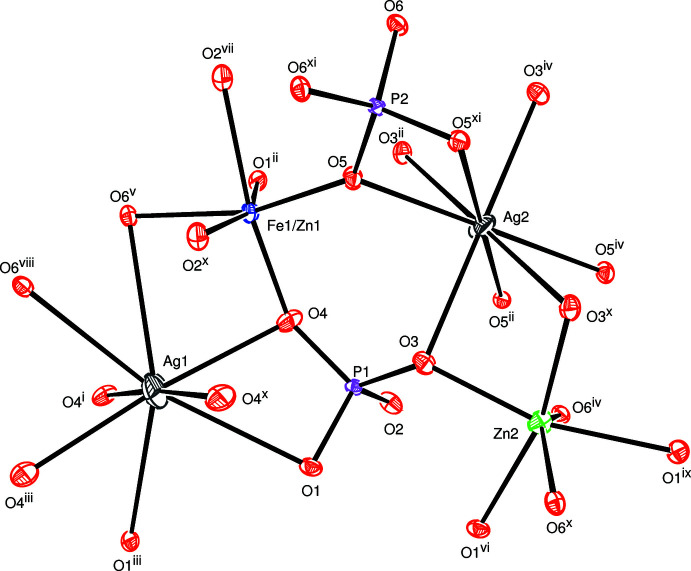
Mol­ecular structure of the title compound with the atom-labelling scheme. Displacement ellipsoids are drawn at the 50% probability level. Symmetry codes: (i) −*x* + 2, *y*, −*z* + 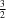; (ii) −*x* + 2, −*y* + 1, −*z* + 1; (iii) *x*, −*y* + 1, *z* + 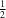; (iv) *x* + 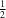, −*y* + 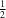, *z* + 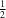; (v) −*x* + 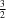, −*y* + 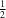, −*z* + 1; (vi) −*x* + 1, −*y* + 1, −*z*; (vii) *x*, −*y* + 1, *z* − 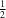; (viii) −*x* + 1, *y*, −*z* + 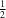; (ix) −*x* + 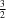, *y* − 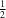, −*z* + 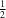; (*x*) −*x* + 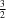, −*y* + 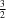, −*z* + 1; (xi) *x* − 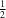, −*y* + 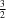, *z* − 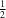.

**Figure 2:**
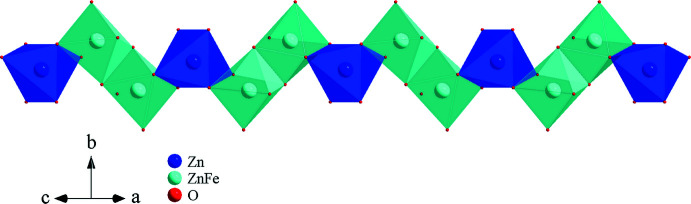
Edge-sharing [(Fe1/Zn1)O_6_] and Zn2O_6_ octa­hedra forming a zigzag chain parallel to the [10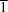] direction.

**Figure 3:**
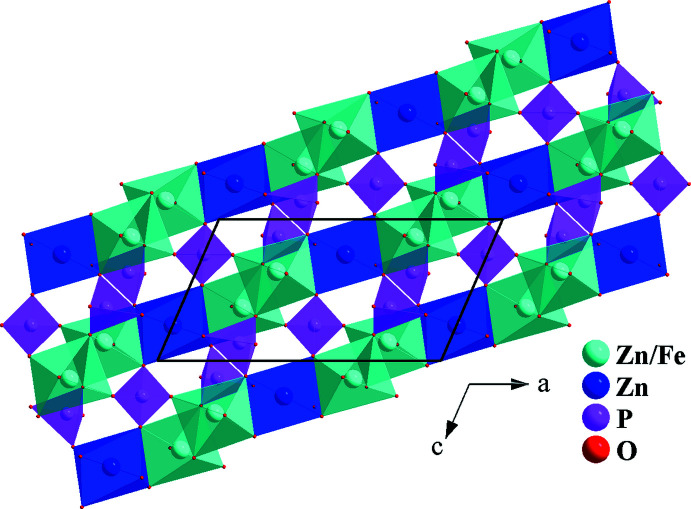
A layer perpendicular to the *b* axis, resulting from the connection of vertices between chains and the PO_4_ tetra­hedra.

**Figure 4:**
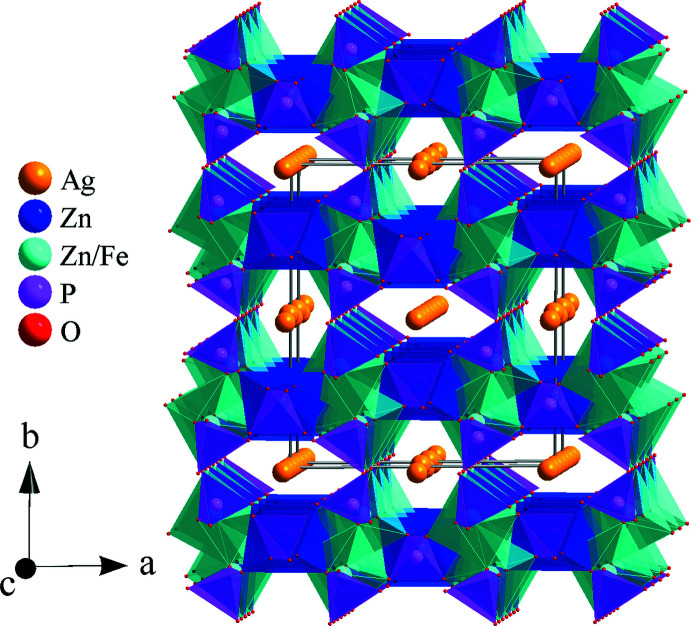
Perspective view of the crystal structure of Ag_1.64_Zn_1.64_Fe_1.34_(PO_4_)_3_, showing the channels running along the [001] direction in which the Ag^+^ are located.

The isolated phosphate, Ag_1.64_Zn_1.64_Fe_1.36_(PO_4_)_3_, crystallizes in the alluaudite structure type. The fundamental building units of the crystal structure are [Ag1O_8_] and [Ag2O_8_] polyhedra, [(Fe1/Zn1)O_6_] and [Zn2O_6_] octa­hedra and two PO_4_ tetra­hedra, as shown in Fig. 1 ▸. In this structure, the Wyckoff position *4e* (twofold) is partially occupied by Ag1 with an occupancy of 64%, while the *4a* (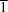) site is entirely occupied by Ag2. The remaining *4e* (twofold) sites are completely filled by P2 and Zn2 atoms. The general position occupied by Fe1/Zn1 exhibits substitutional disorder with statistical distribution of Fe1/Zn1 = 0.68/0.32. The values of the occupancies of these sites were rounded and fixed after the last refinement cycle to respect the electrical neutrality of the structure. The crystal structure consists of extended kinked chains of two edge-sharing [(Fe1/Zn1)O_6_] octa­hedra, leading to the formation of [(Fe1/Zn1)_2_O_10_] dimers. These dimers are connected by a common edge to [Zn2O_6_] units, as depicted in Fig. 2 ▸. Adjacent chains are held together through common vertices with the PO_4_ tetra­hedral groups, to form stacked sheets perpendicular to [010] (Fig. 3 ▸). The resulting three-dimensional framework delimits two types of channel that extend along the [001] direction, hosting Ag^+^ cations (Fig. 4 ▸). Although these cationic sites display the same coordination sphere (CN = 8), their morphologies are clearly different. Indeed, Ag1 adopts a gable disphenoid morphology while Ag2 occupies the centre of a deformed cube. The Ag1—O and Ag2—O inter­atomic distances are in the ranges of 2.495 (2)–2.916 (2) Å, and 2.387 (2)–2.946 (2) Å, respectively. A close examination of effective coordination number (ECoN) for [Ag1]/CN[Ag1] = 7.35/8 *versus* [Ag2]/CN[Ag1] = 6.47/8 ratios reveals a more pronounced distortion in the Ag2O_8_ than in the Ag1O_8_ polyhedra. The mixed-occupancy [Fe1/Zn1] site [occupancy ratio Fe1:Zn1 = 0.68:0.32], is closely surrounded by six oxygen atoms with Fe1/Zn1—O bond lengths ranging from 1.947 (2) Å to 2.246 (2) Å. The second zinc cation Zn2 exhibits a similar coordination sphere with inter­atomic distances varying between 2.091 (2) and 2.198 (2) Å. Both octa­hedral geometries are strongly deformed, with a notable axial compression in [Fe1/Zn1]O_6_ compared to Zn2O_6_. The P—O bond lengths within the regular PO_4_ tetra­hedral units vary between 1.522 (2) and 1.553 (2) Å. Their mean distances <P1—O> = 1.540 Å and <P2—O> = 1.542 Å, are in a good agreement with the <P—O> length usually reported in orthophosphate groups (Baur, 1974 ▸).

## Structural model validation   

In order to support the current crystal structure determination, CHARDI (CHARge-DIstribution) and BVS (Bond-Valence-Sum) analyses were performed using *CHARDI2015* (Nespolo & Guillot, 2016 ▸) and *EXPO2014* (Altomare *et al.*, 2013 ▸) programs, respectively. The results are summarized in Tables 1 ▸ and 2 ▸. For the proposed structural model, BVS were calculated for all constituent atoms using the dual concept: bond lengths/bond strengths. This robust validation method estimates the oxidation states of atoms [valence: *V*(*i*)], evaluates effectively the quality of the crystal structure elucidation and predicts the level of structural strains. In this model, all the nearest ion–counter ion distances less than 3 Å are considered as bonds and taken into account. The CHARDI method is a modern generalization of Pauling’s concept of bond strength (Pauling, 1929 ▸). This approach introduces directly the inter-atomic bond distances in a self-consistent computation to assign a geometrically defined bond strength to each bond. This method adopts a Madelung-type approximation of the crystal structures by attributing point charges to the atoms (the formal charge is equal to the oxidation number; Eon & Nespolo, 2015 ▸). The CHARDI analysis also involves the distribution of computed ECoN of a central atom among all the neighbouring ligands (Hoppe, 1979 ▸). The determination of non-integer ECoN is directly inter­preted in terms of atomic charge distribution in crystalline structures. For a well refined structure, the calculated valences *V*(*i*) and the *Q*(*i*) charges according to BVS and CHARDI concepts must converge towards the weighted oxidation number *q*(*i*)·sof(*i*) of each atom [where *q*(*i*) = formal oxidation number and sof(*i*) = site occupancy]. The resulting values from both conceptions confirm the expected formal ionic charges of Ag^+^, Zn^2+^, Fe^3+^, P^5+^ and O^2−^. In the thirteen independent atomic sites within the asymmetric unit, the cationic charges are located at seven sites, while in the remaining sites the oxygen atoms balance the charges. For all cations, the inter­nal criterion *q*(*i*)/*Q*(*i*) ∼ 1, where *Q*(*i*) represents the computed charge, imply the correctness of the structure determination (Nespolo *et al.*, 1999 ▸). In the structure, all oxygen atoms exhibit a lower over or under bonding (OUB) effect with the exception of atoms O2 and O5, which deviate slightly from the formal value of −2 (Table 1 ▸). To estimate the convergence of the (CHARDI) model, the mean absolute percentage deviation (MAPD) was computed. MAPD measures the agreement between the *q*(*i*) and *Q*(*i*) charges for the whole sets of PC (polyhedron-centring) atoms and of V (vertex) atoms (Nespolo, 2016 ▸), 
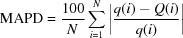
 where *N* is the number of polyhedron-centring or vertex atoms in the asymmetric unit. Respecting this experimental distribution scheme, the resulting values of MAPD for the cationic and anion charges are only 1.1% and 2.4%, respectively. This result supports the applicability and adequacy of the current model.

In order to prove the chemical plausibility of the crystal structure we have also calculated the Global Instability Index (*GII*; Salinas-Sanchez *et al.*, 1992 ▸). The *GII* index 
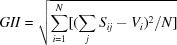
 estimates the coherence of the structure and measures the deviation of the bond-valence sums from the formal valence *V*(*i*) averaged over all N atoms of the asymmetric unit. In our case, we found a very good *GII* index of 0.087 v.u., indicating the stability and the rigidity of the proposed structural model.

## Database survey   

The structure determination of the new phosphate, Ag_1.64_Zn_1.64_Fe_1.36_ (PO_4_)_3_, confirms it to be isotypic with the alluaudite structure. The observed deviation of the chemical formulation from the stoichiometric composition is often encountered in phosphate materials of the alluaudite type *viz*. Na_1.50_Mn_2.48_Al_0.85_(PO_4_)_3_ (Hatert, 2006 ▸), Na_1.25_Mg_1.10_Fe_1.90_(PO_4_)_3_ (Hidouri *et al.*, 2008 ▸), NaFe_3.67_(PO_4_)_3_ (Korzenski *et al.*, 1998 ▸), Na_1.79_Mg_1.79_Fe_1.21_(PO_4_)_3_ (Hidouri *et al.*, 2003 ▸), Na_0.38_Ca_0.31_MgFe_2_(PO_4_)_3_ (Zid *et al.*, 2005 ▸), α**-**Na_0.67_FePO_4_ (Kim *et al.*, 2013 ▸), Li_0.5_Na_0.5_MnFe_2_(PO_4_)_3_ (Trad *et al.*, 2010 ▸), Na_1.5_Mn_1.5_Fe_1.5_(PO_4_)_3_ (Hatert, 2004 ▸), Na_1.86_Fe_3_(PO_4_)_3_ (Essehli *et al.*, 2016 ▸), Na_1.85_Mg_1.85_In_1.15_(PO_4_)_3_&Ag_1.69_Mg_1.69_In_1.31_(PO_4_)_3_ (Ould Saleck *et al.*, 2018 ▸), Ag_1.655_Co_1.647_Fe_1.352_(PO_4_)_3_ (Bouraima *et al.*, 2017 ▸). Generally, in this structure the inter­connected sheets produce two types of hexa­gonal channels parallel to the *c*-axis direction (Hatert, 2008 ▸): channel (1) at (½, 0, *z*) and (0, ½, *z*), while channel (2) is located at (0, 0, *z*) and (½, ½, *z*) (Leroux *et al.*, 1995 ▸). Both channels host two kinds of site: *A*(1) and *A*(2)′. Although *A*(1) and *A*(2)′ are likely to display CN = 8 coordination, they adopt different geometries. For instance in the Ag_1.64_Zn_1.64_Fe_1.36_(PO_4_)_3_ structure, the Ag(2) and Ag(1) cations occupy the *A*(1) and *A*(2)′ sites respectively. However, the morphology of the *A* sites remains a controversial subject. Indeed, Antenucci *et al.* (1995 ▸), brought a restriction on certain cation–oxygen bonds: *A*(1)—O and *A*(2)′—O (*A*—O ∼ 3 Å). Thus the *A* sites can adopt the coordination CN = 6, which implies the passage towards an irregular octa­hedron and deformed trigonal prism for *A*(1) and *A*(2)′, respectively. The evolution from AO_8_ to AO_6_ polyhedra was also reported by Khorari *et al.* (1997 ▸) for a study on the alluaudite NaCaCdMg_2_(AsO_4_)_3_. On the other hand, according to Hatert *et al.* (2006 ▸), the *A*(1) site is distorted cubic, while *A*(2)′ would have a first coordination sphere of only four atoms.

## Synthesis and crystallization   

Single crystals of Ag_1.64_Zn_1.64_Fe_1.36_(PO_4_)_6_ were synthesized by means of a classical solid-state reaction in air. Appropriate amounts of the starting reagents: AgNO_3_, Zn(NO_3_)_2_·6H_2_O, Fe(NO_3_)_3_·9H_2_O, H_3_PO_4_ (85%) were taken in the following molar ratios Ag:Zn:Fe:P = 2:2:1:3. The mixture was dissolved in concentrated nitric acid, stirred at room temperature for 24 h and subsequently evaporated to dryness. The obtained solid was carefully milled in an agate mortar, placed in a platinum crucible and heated up to the melting point of 1223 K. The molten product was maintained at this temperature for 1 h then cooled down slowly to 920 K at rate of 5 K h^−1^ and then rapidly to room temperature by turning off the oven. The title compound was isolated as yellow parallelepiped-shaped crystals.

## Refinement   

Crystal data, data collection and structure refinement details are summarized in Table 3 ▸. The refinement of all the variable parameters leads to well-defined displacement ellipsoids. In the final refinement cycles, the mixed-occupancy (Fe1/Zn1) site was refined with fixed complementary occupancies of 0.68/0.32. This cationic distribution scheme satisfies the electrical neutrality requirement and leads to the corresponding non-stoichiometric compound. The highest peak and the deepest hole in the last difference Fourier map were 0.63 and 0.56 Å from Ag1 and P1, respectively.

## Supplementary Material

Crystal structure: contains datablock(s) I. DOI: 10.1107/S2056989020011408/pk2642sup1.cif

Structure factors: contains datablock(s) I. DOI: 10.1107/S2056989020011408/pk2642Isup2.hkl

CCDC reference: 2024206

Additional supporting information:  crystallographic information; 3D view; checkCIF report

## Figures and Tables

**Table 1:** CHARDI and BVS analysis for the cations in the title compound *q*(*i*) = formal oxidation number; sof(*i*) = site occupancy; CN(*i*) = classical coordination number; *Q*(*i*) = calculated charge; V(*i*) = calculated valence; ECoN(*i*) = effective coordination number.

Cation	*q*(*i*)·sof(*i*)	CN(*i*)	ECoN(*i*)	*V*(*i*)	*Q*(*i*)	*q*(*i*)/*Q*(*i*)
Ag1	0.41	8	6.92	0.82	0.63	1.01
Ag2	1	8	6.47	1.23	0.98	1.02
Fe1/Zn1	2.68	6	5.57	2.67	2.69	1.00
Zn2	2	6	5.91	1.83	2.00	1.00
P1	5	4	3.99	4.94	5.06	0.99
P2	5	4	4.00	4.91	4.89	1.02

**Table 2:** CHARDI calculation for the oxygen anions in the title compound

Anion	*q*(*i*)·sof(*i*)	*Q*(*i*)	*q*(*i*)/*Q*(*i*)
O1	−2	−2.00	1.00
O2	−2	−1.87	1.07
O3	−2	−2.01	1.00
O4	−2	−2.03	0.98
O5	−2	−2.10	0.95
O6	−2	−1.99	1.01

**Table 3:** Experimental details

Crystal data	
Chemical formula	Ag_1.64_Zn_1.64_Fe_1.36_(PO_4_)_3_
*M*_r_	644.97
Crystal system, space group	Monoclinic, *C*2/*c*
Temperature (K)	296
*a*, *b*, *c* (Å)	11.8151 (5), 12.6367 (6), 6.4056 (3)
β (°)	113.431 (2)
*V* (Å^3^)	877.52 (7)
*Z*	4
Radiation type	Mo *K*α
μ (mm^−1^)	10.84
Crystal size (mm)	0.36 × 0.27 × 0.20

Data collection	
Diffractometer	Bruker D8 VENTURE Super DUO
Absorption correction	Multi-scan (*SADABS*; Krause *et al.*, 2015 ▸)
*T*_min_, *T*_max_	0.638, 0.746
No. of measured, independent and observed [*I* > 2σ(*I*)] reflections	25488, 1924, 1585
*R*_int_	0.060
(sin θ/λ)_max_ (Å^−1^)	0.806

Refinement	
*R*[*F* ^2^ > 2σ(*F* ^2^)], *wR*(*F* ^2^), *S*	0.024, 0.044, 1.07
No. of reflections	1924
No. of parameters	95
Δρ_max_, Δρ_min_ (e Å^−3^)	1.41, −0.90

Computer programs: *APEX3* and *SAINT* (Bruker, 2016 ▸), *SHELXT2014/5* (Sheldrick, 2015*a* ▸), *SHELXL2016/6* (Sheldrick, 2015*b* ▸), *ORTEP-3 for Windows* (Farrugia, 2012 ▸), *DIAMOND* (Brandenburg, 2006 ▸) and *publCIF* (Westrip, 2010 ▸).

## supplementary crystallographic information

### Crystal data

**Table:**

Ag_1.64_Zn_1.64_Fe_1.36_(PO_4_)_3_	*F*(000) = 1211
*M**_r_* = 644.97	*D*_x_ = 4.882 Mg m^−^^3^
Monoclinic, *C*2/*c*	Mo *K*α radiation, λ = 0.71073 Å
*a* = 11.8151 (5) Å	Cell parameters from 1924 reflections
*b* = 12.6367 (6) Å	θ = 2.5–35.0°
*c* = 6.4056 (3) Å	µ = 10.84 mm^−^^1^
β = 113.431 (2)°	*T* = 296 K
*V* = 877.52 (7) Å^3^	Parallelepiped, yellow
*Z* = 4	0.36 × 0.27 × 0.20 mm

### Data collection

**Table:**

Bruker D8 VENTURE Super DUO diffractometer	1924 independent reflections
Radiation source: INCOATEC IµS micro-focus source	1585 reflections with *I* > 2σ(*I*)
HELIOS mirror optics monochromator	*R*_int_ = 0.060
Detector resolution: 10.4167 pixels mm^-1^	θ_max_ = 35.0°, θ_min_ = 2.5°
φ and ω scans	*h* = −19→19
Absorption correction: multi-scan (SADABS; Krause *et al.*, 2015)	*k* = −20→20
*T*_min_ = 0.638, *T*_max_ = 0.746	*l* = −10→10
25488 measured reflections	

### Refinement

**Table:**

Refinement on *F*^2^	0 restraints
Least-squares matrix: full	*w* = 1/[σ^2^(*F*_o_^2^) + (0.0142*P*)^2^ + 2.467*P*] where *P* = (*F*_o_^2^ + 2*F*_c_^2^)/3
*R*[*F*^2^ > 2σ(*F*^2^)] = 0.024	(Δ/σ)_max_ = 0.001
*wR*(*F*^2^) = 0.044	Δρ_max_ = 1.41 e Å^−^^3^
*S* = 1.07	Δρ_min_ = −0.90 e Å^−^^3^
1924 reflections	Extinction correction: SHELXL-2018/3 (Sheldrick 2018), Fc^*^=kFc[1+0.001xFc^2^λ^3^/sin(2θ)]^-1/4^
95 parameters	Extinction coefficient: 0.00124 (7)

### Special details

**Table:**

Geometry. All esds (except the esd in the dihedral angle between two l.s. planes) are estimated using the full covariance matrix. The cell esds are taken into account individually in the estimation of esds in distances, angles and torsion angles; correlations between esds in cell parameters are only used when they are defined by crystal symmetry. An approximate (isotropic) treatment of cell esds is used for estimating esds involving l.s. planes.

### Fractional atomic coordinates and isotropic or equivalent isotropic displacement parameters (Å^2^)

**Table:**

	*x*	*y*	*z*	*U*_iso_*/*U*_eq_	Occ. (<1)
Ag1	1.000000	0.49101 (4)	0.750000	0.02841 (12)	0.64
Ag2	0.500000	0.500000	0.000000	0.01591 (7)	
Zn1	0.78254 (3)	0.34690 (2)	0.37235 (5)	0.00670 (6)	0.32
Fe1	0.78254 (3)	0.34690 (2)	0.37235 (5)	0.00670 (6)	0.68
Zn2	0.500000	0.73456 (3)	0.250000	0.01000 (8)	
P1	0.76144 (5)	0.61144 (4)	0.37475 (8)	0.00534 (10)	
P2	0.500000	0.28593 (6)	0.250000	0.00479 (13)	
O1	0.83549 (14)	0.66439 (12)	0.6084 (2)	0.0081 (3)	
O2	0.77834 (14)	0.67712 (12)	0.1861 (3)	0.0096 (3)	
O3	0.62482 (14)	0.60856 (12)	0.3287 (3)	0.0092 (3)	
O4	0.81553 (16)	0.50008 (12)	0.3824 (3)	0.0135 (3)	
O5	0.60368 (13)	0.35972 (12)	0.2534 (3)	0.0084 (3)	
O6	0.45827 (13)	0.21643 (12)	0.0329 (2)	0.0073 (3)	

### Atomic displacement parameters (Å^2^)

**Table:**

	*U*^11^	*U*^22^	*U*^33^	*U*^12^	*U*^13^	*U*^23^
Ag1	0.01096 (18)	0.0277 (3)	0.0355 (3)	0.000	−0.00255 (17)	0.000
Ag2	0.02498 (14)	0.00877 (11)	0.00979 (11)	0.00478 (9)	0.00245 (9)	0.00065 (8)
Zn1	0.00572 (12)	0.00818 (13)	0.00660 (12)	0.00103 (10)	0.00288 (9)	0.00078 (10)
Fe1	0.00572 (12)	0.00818 (13)	0.00660 (12)	0.00103 (10)	0.00288 (9)	0.00078 (10)
Zn2	0.01101 (17)	0.00984 (17)	0.01111 (17)	0.000	0.00646 (14)	0.000
P1	0.0065 (2)	0.0055 (2)	0.0040 (2)	−0.00099 (18)	0.00202 (18)	−0.00037 (18)
P2	0.0051 (3)	0.0052 (3)	0.0036 (3)	0.000	0.0013 (2)	0.000
O1	0.0102 (7)	0.0082 (7)	0.0054 (6)	−0.0012 (5)	0.0024 (5)	−0.0016 (5)
O2	0.0093 (7)	0.0129 (7)	0.0066 (7)	−0.0034 (6)	0.0031 (5)	0.0005 (5)
O3	0.0067 (6)	0.0108 (7)	0.0106 (7)	−0.0015 (5)	0.0039 (6)	−0.0003 (6)
O4	0.0163 (8)	0.0086 (7)	0.0156 (8)	0.0028 (6)	0.0063 (6)	−0.0023 (6)
O5	0.0062 (6)	0.0079 (7)	0.0101 (7)	−0.0005 (5)	0.0021 (5)	0.0023 (5)
O6	0.0061 (6)	0.0086 (7)	0.0062 (6)	−0.0003 (5)	0.0015 (5)	−0.0015 (5)

### Geometric parameters (Å, º)

**Table:**

Ag1—O4	2.4953 (17)	Zn1—O1^vii^	2.0275 (15)
Ag1—O4^i^	2.4953 (17)	Zn1—O2^iii^	2.0525 (15)
Ag1—O4^ii^	2.6368 (18)	Zn1—O6^iv^	2.0762 (15)
Ag1—O4^iii^	2.6368 (18)	Zn1—O2^ix^	2.2463 (16)
Ag1—O1^i^	2.8281 (16)	Zn2—O3	2.0911 (16)
Ag1—O1	2.8282 (16)	Zn2—O3^viii^	2.0911 (16)
Ag1—O6^iv^	2.9160 (15)	Zn2—O6^iii^	2.1497 (15)
Ag1—O6^v^	2.9160 (15)	Zn2—O6^vi^	2.1497 (15)
Ag2—O5^vi^	2.3866 (15)	Zn2—O1^x^	2.1975 (15)
Ag2—O5	2.3866 (15)	Zn2—O1^xi^	2.1975 (15)
Ag2—O3	2.4538 (15)	P1—O3	1.5217 (15)
Ag2—O3^vi^	2.4538 (15)	P1—O4	1.5382 (16)
Ag2—O3^vii^	2.5587 (15)	P1—O2	1.5431 (16)
Ag2—O3^viii^	2.5587 (15)	P1—O1	1.5532 (15)
Ag2—O5^vii^	2.9459 (16)	P2—O5	1.5328 (15)
Ag2—O5^viii^	2.9459 (16)	P2—O5^viii^	1.5328 (15)
Zn1—O5	1.9473 (15)	P2—O6	1.5502 (15)
Zn1—O4	1.9706 (16)	P2—O6^viii^	1.5503 (15)
			
O4—Ag1—O4^i^	174.74 (8)	O5—Zn1—O4	95.79 (7)
O4—Ag1—O4^ii^	102.59 (5)	O5—Zn1—O1^vii^	109.03 (6)
O4^i^—Ag1—O4^ii^	77.17 (5)	O4—Zn1—O1^vii^	88.50 (6)
O4—Ag1—O4^iii^	77.17 (5)	O5—Zn1—O2^iii^	87.17 (6)
O4^i^—Ag1—O4^iii^	102.60 (5)	O4—Zn1—O2^iii^	101.23 (7)
O4^ii^—Ag1—O4^iii^	175.11 (7)	O1^vii^—Zn1—O2^iii^	160.33 (6)
O4—Ag1—O1^i^	119.87 (5)	O5—Zn1—O6^iv^	160.35 (6)
O4^i^—Ag1—O1^i^	55.31 (5)	O4—Zn1—O6^iv^	102.51 (6)
O4^ii^—Ag1—O1^i^	61.28 (5)	O1^vii^—Zn1—O6^iv^	78.85 (6)
O4^iii^—Ag1—O1^i^	114.48 (5)	O2^iii^—Zn1—O6^iv^	82.34 (6)
O4—Ag1—O1	55.31 (5)	O5—Zn1—O2^ix^	77.79 (6)
O4^i^—Ag1—O1	119.87 (5)	O4—Zn1—O2^ix^	171.76 (6)
O4^ii^—Ag1—O1	114.48 (5)	O1^vii^—Zn1—O2^ix^	88.76 (6)
O4^iii^—Ag1—O1	61.28 (5)	O2^iii^—Zn1—O2^ix^	83.75 (6)
O1^i^—Ag1—O1	78.45 (6)	O6^iv^—Zn1—O2^ix^	84.57 (6)
O4—Ag1—O6^iv^	70.89 (5)	O3—Zn2—O3^viii^	80.82 (8)
O4^i^—Ag1—O6^iv^	114.20 (5)	O3—Zn2—O6^iii^	113.09 (6)
O4^ii^—Ag1—O6^iv^	83.66 (5)	O3^viii^—Zn2—O6^iii^	92.67 (6)
O4^iii^—Ag1—O6^iv^	100.79 (5)	O3—Zn2—O6^vi^	92.67 (6)
O1^i^—Ag1—O6^iv^	144.45 (4)	O3^viii^—Zn2—O6^vi^	113.09 (6)
O1—Ag1—O6^iv^	125.39 (4)	O6^iii^—Zn2—O6^vi^	146.50 (8)
O4—Ag1—O6^v^	114.20 (5)	O3—Zn2—O1^x^	85.40 (6)
O4^i^—Ag1—O6^v^	70.89 (5)	O3^viii^—Zn2—O1^x^	164.84 (6)
O4^ii^—Ag1—O6^v^	100.79 (5)	O6^iii^—Zn2—O1^x^	86.93 (6)
O4^iii^—Ag1—O6^v^	83.66 (5)	O6^vi^—Zn2—O1^x^	73.66 (5)
O1^i^—Ag1—O6^v^	125.39 (4)	O3—Zn2—O1^xi^	164.84 (6)
O1—Ag1—O6^v^	144.45 (4)	O3^viii^—Zn2—O1^xi^	85.40 (6)
O6^iv^—Ag1—O6^v^	51.96 (6)	O6^iii^—Zn2—O1^xi^	73.66 (5)
O5^vi^—Ag2—O5	180.0	O6^vi^—Zn2—O1^xi^	86.93 (6)
O5^vi^—Ag2—O3	98.00 (5)	O1^x^—Zn2—O1^xi^	108.94 (8)
O5—Ag2—O3	82.00 (5)	O3—Zn2—O5^vi^	84.84 (5)
O5^vi^—Ag2—O3^vi^	82.00 (5)	O3^viii^—Zn2—O5^vi^	61.40 (5)
O5—Ag2—O3^vi^	98.00 (5)	O6^iii^—Zn2—O5^vi^	146.63 (5)
O3—Ag2—O3^vi^	180.0	O6^vi^—Zn2—O5^vi^	51.69 (5)
O5^vi^—Ag2—O3^vii^	109.52 (5)	O1^x^—Zn2—O5^vi^	123.73 (5)
O5—Ag2—O3^vii^	70.48 (5)	O1^xi^—Zn2—O5^vi^	83.04 (5)
O3—Ag2—O3^vii^	114.55 (6)	O3—Zn2—O5^iii^	61.40 (5)
O3^vi^—Ag2—O3^vii^	65.45 (6)	O3^viii^—Zn2—O5^iii^	84.84 (5)
O5^vi^—Ag2—O3^viii^	70.48 (5)	O6^iii^—Zn2—O5^iii^	51.69 (5)
O5—Ag2—O3^viii^	109.52 (5)	O6^vi^—Zn2—O5^iii^	146.63 (5)
O3—Ag2—O3^viii^	65.45 (6)	O1^x^—Zn2—O5^iii^	83.04 (5)
O3^vi^—Ag2—O3^viii^	114.55 (6)	O1^xi^—Zn2—O5^iii^	123.73 (5)
O3^vii^—Ag2—O3^viii^	180.0	O5^vi^—Zn2—O5^iii^	136.14 (5)
O5^vi^—Ag2—O5^vii^	53.05 (6)	O3—P1—O4	112.31 (9)
O5—Ag2—O5^vii^	126.95 (6)	O3—P1—O2	108.59 (9)
O3—Ag2—O5^vii^	83.55 (5)	O4—P1—O2	109.61 (9)
O3^vi^—Ag2—O5^vii^	96.45 (5)	O3—P1—O1	110.16 (9)
O3^vii^—Ag2—O5^vii^	70.07 (5)	O4—P1—O1	107.20 (9)
O3^viii^—Ag2—O5^vii^	109.93 (5)	O2—P1—O1	108.92 (9)
O5^vi^—Ag2—O5^viii^	126.95 (6)	O5—P2—O5^viii^	105.07 (12)
O5—Ag2—O5^viii^	53.05 (6)	O5—P2—O6	108.94 (8)
O3—Ag2—O5^viii^	96.45 (5)	O5^viii^—P2—O6	111.39 (8)
O3^vi^—Ag2—O5^viii^	83.55 (5)	O5—P2—O6^viii^	111.39 (8)
O3^vii^—Ag2—O5^viii^	109.93 (5)	O5^viii^—P2—O6^viii^	108.94 (8)
O3^viii^—Ag2—O5^viii^	70.07 (5)	O6—P2—O6^viii^	110.98 (12)
O5^vii^—Ag2—O5^viii^	180.0		

Symmetry codes: (i) −*x*+2, *y*, −*z*+3/2; (ii) −*x*+2, −*y*+1, −*z*+1; (iii) *x*, −*y*+1, *z*+1/2; (iv) *x*+1/2, −*y*+1/2, *z*+1/2; (v) −*x*+3/2, −*y*+1/2, −*z*+1; (vi) −*x*+1, −*y*+1, −*z*; (vii) *x*, −*y*+1, *z*−1/2; (viii) −*x*+1, *y*, −*z*+1/2; (ix) −*x*+3/2, *y*−1/2, −*z*+1/2; (x) −*x*+3/2, −*y*+3/2, −*z*+1; (xi) *x*−1/2, −*y*+3/2, *z*−1/2.
